# Degradation of Metal-Organic Framework Materials as Controlled-Release Fertilizers in Crop Fields

**DOI:** 10.3390/polym11060947

**Published:** 2019-06-01

**Authors:** Ke Wu, Changwen Du, Fei Ma, Yazhen Shen, Dong Liang, Jianmin Zhou

**Affiliations:** 1 The State Key Laboratory of Soil and Sustainable Agriculture, Institute of Soil Science Chinese Academy of Sciences, Nanjing 210008, China; kwu@issas.ac.cn (K.W.); fma@issas.ac.cn (F.M.); yzshen@issas.ac.cn (Y.S.); dliang@issas.ac.cn (D.L.); jmzhou@issas.ac.cn (J.Z.); 2 College of Advanced Agricultural Sciences, University of Chinese Academy of Sciences, Beijing 100049, China

**Keywords:** degradation, metal-organic frameworks, controlled-release fertilizer, field wheat soil

## Abstract

The behavior of a metal-organic framework (MOF) compound synthesized in hydrothermal reaction conditions and rich in N, P, and Fe nutrients was explored in the field. The attenuated total reflection-Fourier transform infrared (ATR-FTIR) spectroscopy and laser induced breakdown spectroscopy (LIBS) characterization results showed that the chemical structures changed during the degradation process in crop field soil. The scanning electron microscope images showed that the micro-rod of the MOF peeled off and degraded in layers. During the growth period of wheat, the MOF degraded by 50.9%, with the degradation rate being closely related to soil temperature. It was also found that the degradation rate increased with soil temperature. Moreover, the nutrient concentration of the soil indicated that the MOF had stable nutrients release efficiencies and could provide a continuous supply of nutrients throughout the wheat growth period, which showed a great alternative for MOF as a fertilizer both benefiting agricultural production and environmental protection.

## 1. Introduction

Fertilizers play an important role in agricultural production. According to statistical data by the Food and Agriculture Organization (FAO) of the United Nations, fertilizer accounts for a 40–60% increase in grain yields [1]. At present, the world’s population is approximately 7 billion, and it is expected to reach 9.5 billion by 2050 [2]. At that time, the demand for food will be twice what it is today. It can be foreseen that chemical fertilizers will become more prominent in the coming decades, and the amount of input will also increase substantially [3].

Nitrogen and phosphorus are essential nutrients for plant growth and development. Globally, nitrogen fertilizers have played an irreplaceable role in improving crop yields and farmers’ economic returns [4]. Since the first green revolution, the application of nitrogen fertilizers has greatly increased the grain yield per hectare. Between 1960 and 1995, the world’s grain production increased by 100%, while the amount of nitrogen fertilizer increased by a factor of 6.9 [5,6]. However, excessive utilization of nitrogen fertilizers has caused a series of problems, such as a waste of resources and several environmental issues. When nitrogen fertilizers are applied to soil, three flow paths are possible: absorption by plants, accumulation in the soil, and loss through leaching and volatilization [7]. Studies have shown that nitrogen leaching and volatilization are closely related to the eutrophication of water bodies and greenhouse gas emissions [8,9]. Relevant research results show that direct and indirect losses caused by the loss of nitrogen to the environment are as high as 100 million euros per year [10].

Phosphorus plays an important role in agricultural production. However, the utilization rate of a phosphate fertilizer is usually only 10–25% [11], resulting in a surplus of phosphorus nutrients in farmland. This continuous accumulation of soil phosphorus increases risks, such as possible water ecosystem imbalances and various types of water pollution problems, for the environment. It is generally believed that the main source of phosphorus in farmlands is the loss of surface runoff. However, owing to the strong ability of the soil to fix phosphorus, it is unlikely that there will be phosphorus leaching [12]. However, excessive phosphorus accumulation in the soil increases the probability of leaching [13]. Therefore, the phosphorus level in soil is an important factor affecting the loss of phosphorus from farmlands.

Methods to increase the absorption and utilization of nitrogen and phosphorus in crops, thereby reducing losses and environmental pollution, have always been a focus of research in this field. Some traditional methods include suitable fertilizer application rates, fertilizer and water control techniques, fractional fertilization, and balanced fertilization. There are also new methods involving real-time monitoring of field nitrogen fertilizers and precise management techniques for farmland nutrients. These methods can theoretically improve the utilization of fertilizers and reduce environmental risks. However, traditional methods not only increase the labor cost, but also make promotion difficult. Further, the new methods are still in the developmental stages and have many limitations. The application of new fertilizers, especially controlled-release fertilizers, as one of the important ways to increase the utilization rate of fertilizers, has received extensive attention in recent decades.

Metal-organic frameworks (MOFs) area type of material with adjustable pore sizes formed by the self-assembly of organic ligands coordinated into metal ions or clusters. MOFs have been used in many fields, such as gas storage [14,15,16], catalysis [17] and as drug carriers [18,19,20], and its application as fertilizers has been reported. The impact of synthesized oxalate-phosphate-amine metal-organic-frameworks (OPA-MOFs) on the growth, nutrient uptake and grain yield of wheat were investigated. The results showed that the OPA-MOF has a potential as enhanced efficiency N fertilizer [21,22]. Additionally, iron-metal-organic-frameworks-ethylenediaminetetra-acetic acid (Fe-MOF-EDTA) was used as Fe-sources in Phaseolus vulgaris, in comparison with other Fe-sources, the Fe-MOF-EDTA caused a 9.6% enhancement in plant weight and improvement of chlorophyll content, protein and enzymes activities [23]. These previous studies have verified the potential of MOF as fertilizers. Therefore, we attempted to synthesize a novel MOF and explore its potential as fertilizer. For application as fertilizers, MOFs should contain the elements needed by crops as nutrients, such as nitrogen, phosphorus, and possibly essential metal micro-nutrients, such as iron, zinc, etc. For the inorganic portion of MOFs, iron-phosphates have shown great potential, and have been applied in many fields [24,25,26], with zinc added as a trace element; for the organic ligands, oxalate shows strong coordination ability, especially with transition metals [27]. Oxalate is a dicarboxylic acid and plays an important role in soil. Studies have shown that oxalate can increase the P bio-availability in soil [28]. Oxalotrophic bacteria, a group of bacteria existing in almost all types of soils, use oxalate as a carbon source to satisfy their energy metabolism requirements, resulting in the formation of carbonates, which is a metabolic process known as the oxalate-carbonate pathway [29,30]. Structure-directing agents (SDAs) are used to obtain more stable target products, larger possible pore sizes, and increased surface areas inside the pores. Amines, especially di-amines, such as diaminopropane, and piperazines, have become useful as SDAs [31,32,33]. In general, SDAs remain largely unchanged and are present as guests in the framework’s pores. In some cases, SDAs fully or partially decompose into a more stable secondary structure [34].

In this study, a novel MOF compound was synthesized under mild hydrothermal conditions with urea as an SDA, an iron-phosphate backbone as the inorganic portion, and oxalic acid as the organic ligand. The degradation and nutrient release processes of the MOF were investigated by some novel instrumental techniques; and the potential of the MOF was then assessed as controlled-release fertilizers.

## 2. Materials and Methods

### 2.1. Synthesis of MOF

A novel MOF was synthesized under mild hydrothermal conditions. The raw materials included ferric chloride (FeCl_3_∙6H_2_O), phosphoric acid (H_3_PO_4_), oxalic acid (H_2_C_2_O_4_∙2H_2_O), urea (CO(NH_2_)_2_), and de-ionized water (H_2_O). The substrate molar ratios are as follows: ferric chloride:phosphoric acid:oxalic acid:urea:de-ionized water = 1:6:1:2.5:100. The mixture was transferred into a Teflon container (KCF-2, Beijing Century Senlang experimental apparatus Co., Ltd., Beijing, China), and maintained at 100 °C for 24 h. Three replicates of the solution were prepared. All reagents were purchased from Nangjing Ronghua Scientific Equipment Co., Ltd. (Nangjing, China), and all reagents used in the syntheses were of analytical grade. The obtained solids were washed three times with de-ionized water and dried at 60 °C, then sieved to 3 mm. According to the determination, the MOF nutrient contents are as follows: N (5.16%), P (14.68%), and Fe (18.56%).

### 2.2. Powder X-ray Diffraction (PXRD) Characterization of MOF

PXRD data were collected in the 3–70° range by the ARL X’TRA diffractometer (Thermo Electron Corporation, Ecublens, Switzerland) using CuKα radiation source at 0.02° step size and 5°/min scanning rate, and the powder XRD data of MOF was compared to the International Centre for Diffraction Data (ICDD) for phase identification.

### 2.3. Experiment Design

Our field trials were conducted at the Tangquan Experimental Base of the Nanjing Institute of Soil Science, Chinese Academy of Sciences (32°04′15″ N, 118°28′21″ E) in Jiangsu province, China. The basic physicochemical properties of soil are shown in Table 1.

A 2.5 g sample of MOF was wrapped in nylon gauze with an aperture of 74 microns, and record it as a material sample. A total of 16 samples were designed and prepared using this method and were buried 15 cm from the soil horizon when sowing wheat. Two adjacent samples were placed 15cm apart from each other. Samples of the materials and the soil near to the nylon gauze were taken on the first, second, fourth and sixth months. Four MOF samples and four soil samples were taken each time. The background soil was assumed as the control. The MOF material samples were cleaned and dried at 60 °C for structure characterization, and weighed it for calculation of degradation rate, while the soil samples were dried naturally for nutrient determination.

### 2.4. Attenuated Total Reflection-Fourier Transform Infrared (ATR-FTIR) Spectroscopy Characterization

Attenuated total reflection-Fourier transform infrared spectroscopy (ATR-FTIR) (4300 Handheld FTIR, Agilent Technologies, Palo Alto, CA, USA) was used to record the spectrum of the MOF samples. Scans were performed in the range of 4000–650 cm^−1^, the resolution was 4 cm^−1^, and the background and each sample were scanned eight times in succession.

### 2.5. Laser Induced Breakdown Spectroscopy (LIBS) Characterization

A MobiLIBS system (IVEA, France) was used for laser-induced breakdown spectroscopy (LIBS) to determine the atomic composition and content of the material samples. The system consisted of a fourth-harmonic Nd:YAG laser (Quantel, Paris, France) driving 5-ns pulses. The frequency, delivery energy, and wavelength of the pulsed laser were 20 Hz, 16 mJ, and 266 nm (Nd-YAG), respectively. In this system, in the spectral range of 200–1000 nm, an intensified charge-coupled device camera (iStar, Andor Technology, Ltd., Belfast, Northern Ireland) was used to collect the diffracted light. We set 2 × 2 matrices for each shot.

### 2.6. Scanning Electron Microscopy (SEM) Characterization

The micro-structure of MOF material was observed more intuitively using scanning electron microscopy (SEM, MERLIN Compact, Carl Zeiss AG, Oberkochen, Germany) on 10 kV accelerating voltage (AV), to characterize its morphology and surface topography.

### 2.7. Powder X-ray Diffraction (PXRD) Characterization of MOF Samples

PXRD data of MOF samples at different degradation stages were collected in the 3–70° range by the ARL X’TRA diffractometer (Thermo Electron Corporation, Ecublens, Switzerland) using CuKα radiation source at a 0.02° step size and a 5°/min scanning rate.

### 2.8. Determination of Soil Nutrient

In order to explore the nutrient release status of the MOF material, the soil samples nutrients were determined. The content of NO_3_^−^-N and NH_4_^+^-N were determined by using SmartChem200 discrete auto analyzer (AMS Alliance, Frepillon, France). The available P and available Fe were determined by iCAP-7000 ICP-OES spectrometer (Thermo Fisher Scientific, Waltham, MA, USA). A pH meter (Orion Star A211, Thermo Fisher, Scientific, Waltham, MA, USA) was used to determine the soil pH. At the same time, the weather station (WatchDog2000 series, Spectrum, Chicago, IL, USA) recorded daily weather patterns, soil water content, and soil temperature.

## 3. Results

### 3.1. PXRD Characterization of MOF

**Figure 1:**
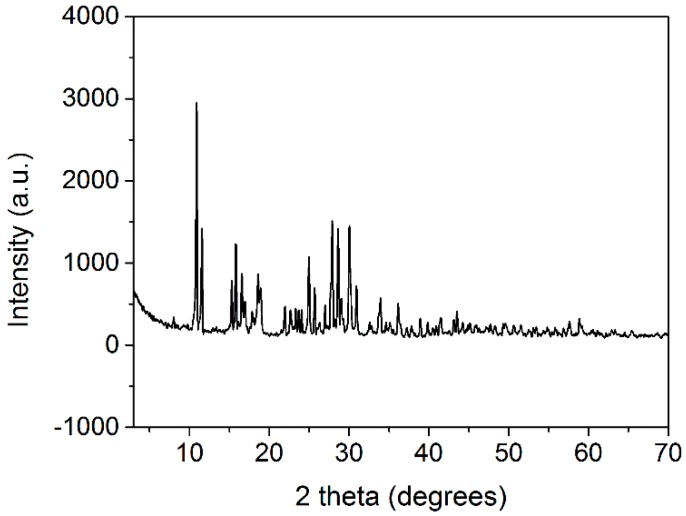
PXRD of MOF.

As shown in Figure 1, the PXRD pattern of MOF was very different from the previous study [21]. Furthermore, in comparison to the International Centre for Diffraction Data (ICDD), since no match was found, it was assumed that our MOF was a newly synthesized compound.

### 3.2. ATR-FTIR Characterization

**Figure 2:**
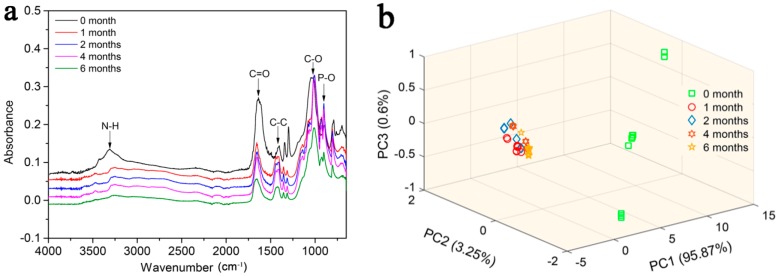
Average ATR-FTIR spectra of MOF and the principal component analysis (PCA) distributions based on the ATR-FTIR of the MOF with different treatments. (**a**). Spectra of MOF degraded in wheat farm soil for zero months, one month, two months, four months, and six months. (**b**). The first three PCA scatterplots based on the ATR-FTIR spectra of MOF.

The average ATR-FTIR spectral shapes and characteristic absorption peaks of the MOF samples are similar for different degradation periods (Figure 2a); the broad peak in the range of 3500–3000 cm^−1^ can be associated with the N–H stretching vibration and the peaks at approximately 1662 cm^−1^ and 1431 cm^−1^ correspond to C=O and C–C stretching vibrations, respectively. Regarding the fingerprint area, the peaks at 1022 and 902 cm^−1^ were mainly associated with C–O and P–O stretching vibrations, respectively. However, the intensity of the characteristic absorption peaks varied greatly for different treatments. The absorbance intensity at zero months is significantly greater than other treatments, which may indicate breaks in the chemical bonds during the degradation of the compound, such as C=O, C–C, and C–O bonds. Principal component analysis (PCA) was used to reduce the dimensions of the spectral data by providing new variables. The principal component distributions based on the FTIR-ATR of the MOF are shown in Figure 2b. The first three principal components (PC1), (PC2), and (PC3) accounted for 99.72% of the total variance. Therefore, PC1, PC2, and PC3 can accurately reflect information regarding the original variables. In Figure 2b, the scores of PC2 and PC3 show irregular distributions for all treatments. Also, it is easy to distinguish treatment at 0 month from the other treatments using the distribution of the PC1 scores, indicating a chemical change in the compounds during the degradation process. However, the PC1, PC2, and PC3 still show irregular distributions for the one month, two months, four months, and six months treatments.

### 3.3. LIBS Characterization

**Figure 3:**
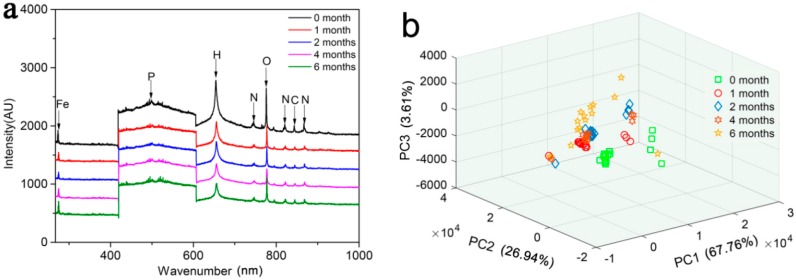
Average LIBS spectra of MOF and the principal component analysis (PCA) distributions based on the LIBS of the MOF with different treatments. (**a**). Spectra of MOF degraded in wheat farm soil for zero months, one month, two months, four months, and six months. (**b**). The first three PCA scatterplots based on the LIBS of MOF.

The average LIBS of the MOF samples at different degradation stages are shown in Figure 3a. There is almost no change in the atomic emission peak positions of the MOF. The following spectral signals were used to characterize the samples: Fe (274.6 nm), P (500.3 nm), C (844.8 nm), N (746.8, 819.2, 868.3 nm), O (777.3 nm), and H (655.6 nm). However, the intensity of the spectral absorption peak gradually weakened with the degradation progress, especially for the N and H signals. Our unpublished results about single crystal analysis results showed that the N nutrition is located inside the MOF as NH^4+^. During the degradation of the MOF material, the framework structure gradually collapses, resulting in the release of the N nutrients embedded in the skeleton; perhaps this explains the decrease in the N and H element characteristic peaks. Principal component distributions based on the LIBS of the MOF are shown in Figure 3b. The PC1, PC2, and PC3 explained the variances of 98.31%, which could be used to represent their spectral variations. The scores of PC1 show irregular distributions for the treatments. However, the zero month, two months, and six months treatments are easy to distinguish using the score of PC2, indicating that the content of MOF elements has changed during the degradation process.

### 3.4. SEM Characterization

**Figure 4:**
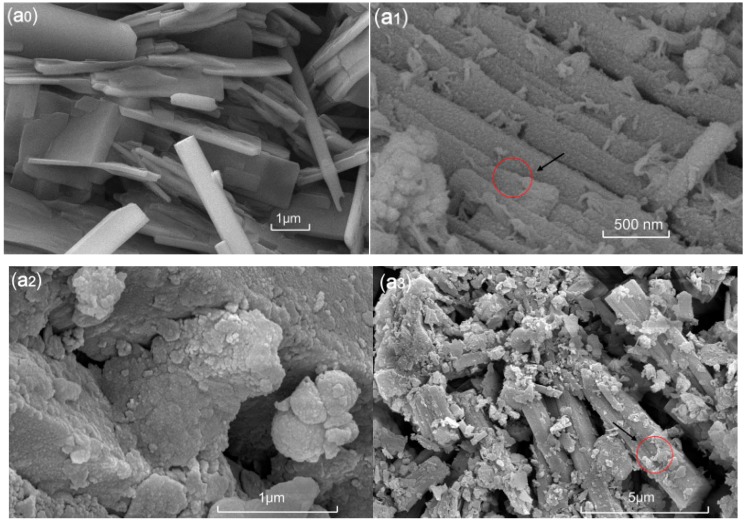

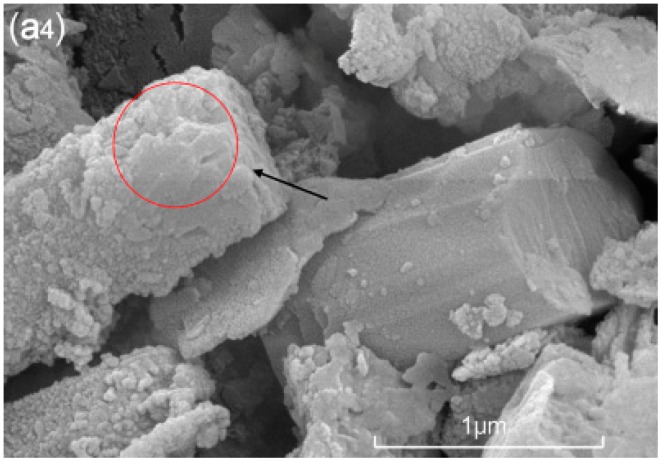
SEM micrographs of the MOF samples (**a_0_**,**a_1_**,**a_2_**,**a_3_**,**a_4_**) at different degradation times in the wheat farm soil. **a_0_**, zero months; **a_1_**, one month; **a_2_**, two months; **a_3_**, four months; and **a_4_**, six months.

The micrograph of MOF (Figure 4a_0_) reveals that a rod-like microstructure with rods of many different lengths aggregated. Most of the rods have different dimensions, with lengths of approximately 1–10 μm, and diameter range of 100–200 nm. Interestingly, the surfaces of these micro-rod structures are very smooth. In the subsequent month or two, the surface becomes rough, and the microstructure of the surface begins to disintegrate (Figure 4(a_1_,a_2_)). With time, the surface structure of the micro-rod peeled off and degraded in layers (Figure 4(a_3_,a_4_)).

### 3.5. PXRD Characterization of MOF Samples

**Figure 5:**
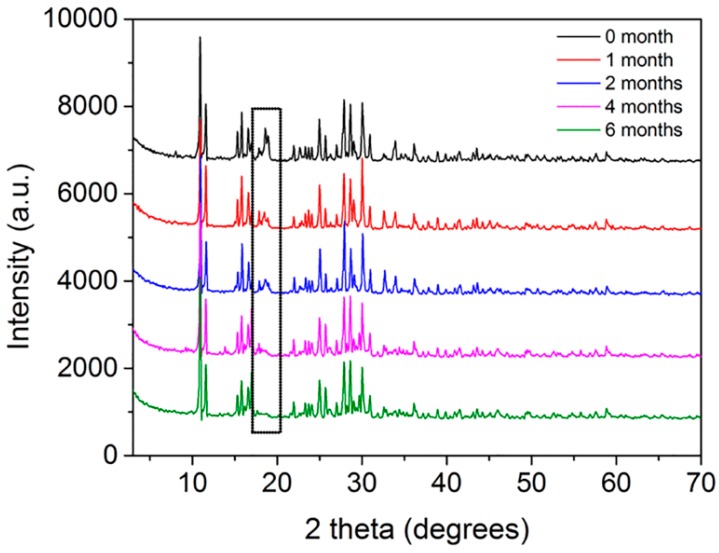
PXRD of MOF samples at different degradation stages.

As shown in Figure 5, the characteristic peak intensity at 18.6° gradually decreased with the degradation. Besides, the crystallinity of MOF samples at 0, 1, 2, 4, and 6 months are 96.64%, 91.09%, 89.70%, 86.01, and 82.23%, respectively, indicating that the longer the degradation time, the lower the crystallinity. These results confirmed that the crystal structure was destroyed during the MOF degradation process.

### 3.6. Nutrient Release Status of the MOF

**Figure 6:**
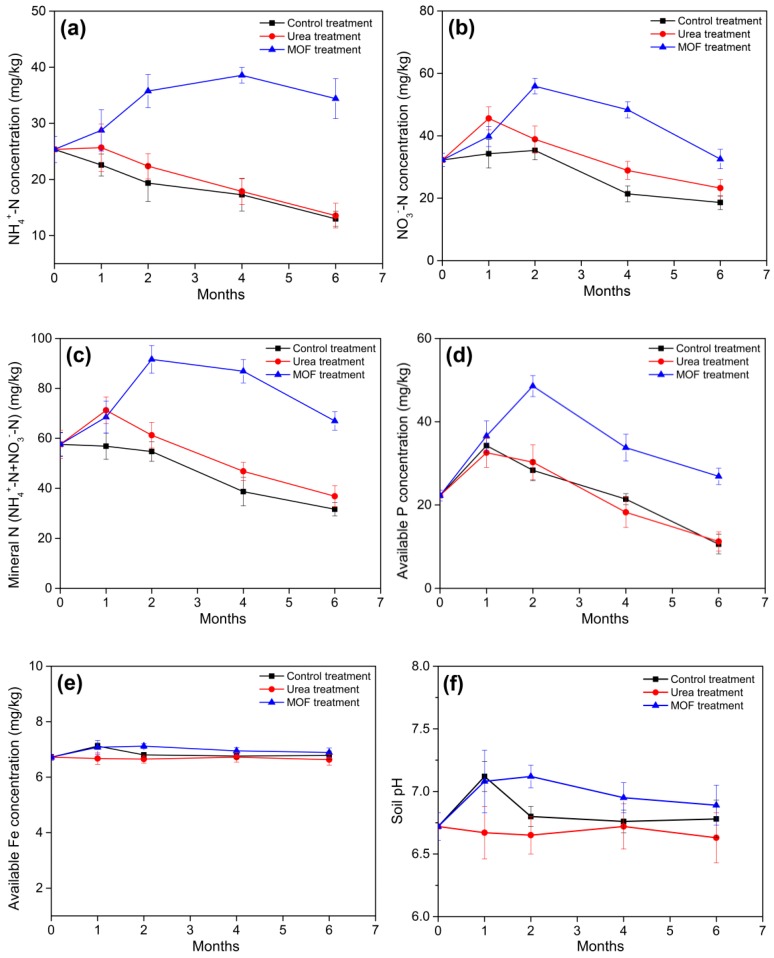
Effects of the MOF on soil nutrients. (**a**) NH_4_^+^-N; (**b**) NO_3_^−^-N; (**c**) mineral N (NH_4_^+^-N + NO_3_^−^-N); (**d**) available P; (**e**) available Fe; and (**f**) soil pH.

In the control and Urea treatments, the NH_4_^+^-N concentration continued to decline throughout the wheat growing period. However, for the MOF treatment, theNH_4_^+^-N concentration increased continuously in the first four months, then began to decline slowly; after six months, the NH_4_^+^-N concentration was still much higher than that in the other two treatments (Figure 6a). Compared with control and urea treatments, the application of the MOF resulted in a very large increase in soil NO_3_^−^-N, mineral N and available P concentration, and reached maximums at two months, then declined (Figure 6b–d), which is probably due to the increased demand of nutrients for the wheat. In contrast to the control and urea treatments, the soil mineral N and available P concentration remained higher even after six months. However, the available Fe concentration did not differ greatly among the three treatments (Figure 6e). Additionally, the MOF also have an affected the soil pH, the soil pH increased in the first two months, before decreasing slowly. After 6 months, the pH was still slightly higher than that in the other two treatments, implying that the degradation of the compound can increase soil pH (Figure 6f). 

### 3.7. Effects of Soil Temperature and Soil Water Content on the Degradation of the MOF

**Figure 7:**
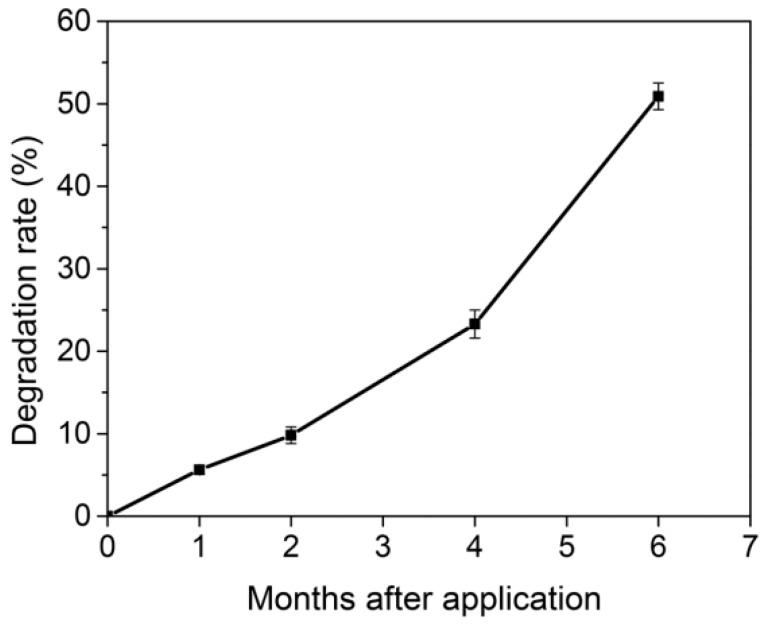
Degradation rate of MOF.

**Figure 8:**
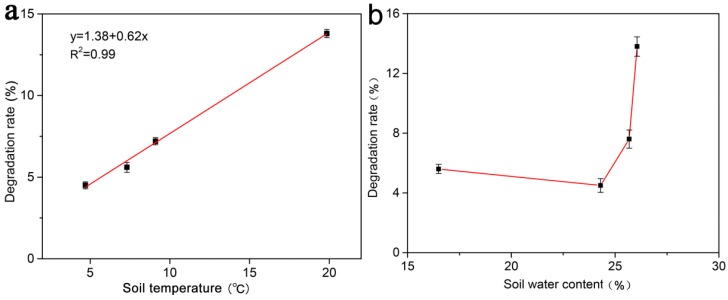
Effects of soil temperatures and soil water contents on the degradation of the MOF over a month. (**a**). Effects of soil temperatures on the degradation of MOF. (**b**). Effects of soil water contents on the degradation of content.

In the six months of the wheat season, the MOF degraded by 50.9% (Figure 7), the degradation rate in one month differed due to many factors. The results showed that the effect of temperature on the degradation rate was extremely significant (Figure 8a). When the average soil temperature was 4.7 °C, the degradation rate of the MOF in one month was 4.2%. However, when the average soil temperature was 19.8 °C, the degradation rates in one month reached 13.8%. The degradation rate had a linear relationship with temperature, i.e., the higher the temperature was, the faster the degradation was. The correlation coefficient (R^2^) between the degradation rate and the temperature reached 0.99, which indicates that the MOF degradation rate was greatly affected by temperature. However, the effect of soil water content on the degradation rate of the MOF seems to be irregular (Figure 8b). In this instance, with average soil water content in the range of 16.5–26.1%, it may not have been the main factor affecting the degradation rate. Furthermore, soil is a complex system where soil physics, chemistry, biology, and other factors may affect the MOF degradation.

## 4. Discussion

### 4.1. Mechanism of Degradation of the MOF

**Figure 9:**
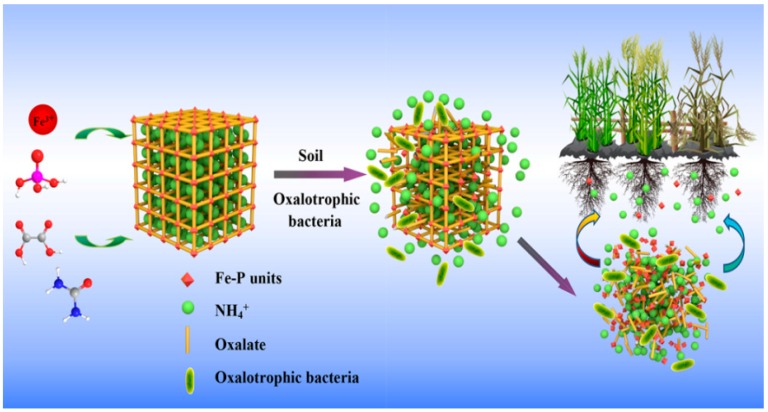
Schematic diagram of the MOFs’ degradation.

The degradation process of the MOF is shown in Figure 9. In this study, oxalic acid was selected as the organic ligand due to its role in the soil ecosystem. It has been reported that oxalate plays an important role in increasing the soil available phosphorus [35,36]. The oxalate-carbonate pathway, which is considered an important part of the biochemical carbon cycle on Earth. The essence of this pathway is the transformation of oxalate to carbonate, usually a bio-transformation of calcium oxalate to calcium carbonate, resulting in an increase in the soil pH. However, spontaneous oxidation of oxalate, especially in the case of calcium oxalate, is unlikely to occur due to the high activation energy required [37]. Further, the currently known existing microorganisms can drive this pathway using oxalotrophy, where a group of bacteria use oxalate as carbon and energy sources. These bacteria have been demonstrated by inoculating agar plates with enriched soil solutions. Calcium oxalate-modified agar has an opaque appearance due to the low solubility of calcium oxalate. Therefore, the clarification zone surrounding calcium oxalate particles shows the consumption of calcium oxalate by bacterial colonies growing on the plate. This further proves the existence of oxalate consuming bacteria in the soil [38]. Therefore, these bacteria may cause the degradation of MOF by consuming oxalate.

### 4.2. MOFs as Controlled-Release Fertilizer

**Figure 10:**
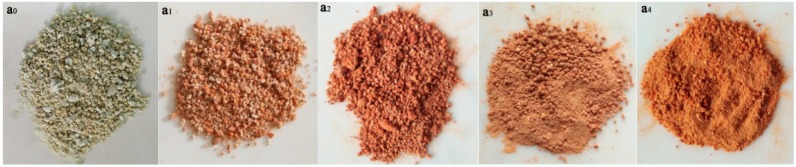
Morphology of MOF (**a_0_**,**a_1_**,**a_2_**,**a_3_**,**a_4_**) at different degradation times in the wheat farm soil. **a_0_**, zero month; **a_1_**, one month; **a_2_**, two months; **a_3_**, four months; **a_4_**, six months.

A novel MOF was successfully synthesized under hydrothermal reaction conditions. A hydrothermal reaction is a relatively mild synthetic process where each reaction parameter is easy to control. The substrates used to synthesize the target compounds are non-toxic, inexpensive, and easily obtained; therefore, the compounds are environmentally friendly and exhibit the possibility of industrial production. Furthermore, the nutrient content of MOF is N, 5.16% and P, 14.68%, which contains more nutrient than those previous synthesized MOF material (N, 3.1% and P, 12.5%) [22]. Therefore, the nutrient content provides a theoretical basis for its potential application as controlled-release fertilizers. The SEM micrographs of the different degradation stages of MOF in the soil clearly demonstrate that the MOF degraded and peeled off in layers (Figure 4). Additionally, the MOF materials gradually change from yellow to brownish red during the degradation process (Figure 10), which implies the likely occurrence of biological oxidation.

In six months, the MOF degraded by 50.9%. In the first month, it degraded by 5.6%, but it only degraded by 4.2% in the second month, which is presumably due to the low soil temperature. The average soil temperature was 7.3 °C in the first month, while the second month was in the winter and the snowfall was relatively large, resulting in an average soil temperature of only 4.7 °C. As the temperature increased, the degradation rate of the compound gradually increased. The degradation rate and temperature were highly correlated, suggesting that temperature was the main factor affecting degradation. It was well known that temperature has a great influence on soil microbial activity, and that microbial activity generally increases with increasing temperature. Although the soil water content also changes, its effect on degradation is not regular; thus, it may not be a major factor affecting degradation (Figure 8b). In the first four months, for treatment with the application of MOF, the NH_4_^+^-N concentration in soil increased due to the continuous release of ammonium ions from the MOF compound (Figure 6a). NH_4_^+^-N was converted to NO_3_^−^-N due to nitrification, resulting in an increase in the NO_3_^−^-N concentration in the first two months. With the increased demand for nutrients during the growth of wheat, the mineral N and available P concentration began to decline; however, the final concentration was still higher than that in the control and urea treatments (Figure 6b–d). These results indicate that the MOF can release nutrients continuously and steadily, which is consistent with previous research [22]. Meanwhile, the differences in the available Fe contents in the treated soils were not large. In fact, soil available Fe accounted only for a small part of the soil total Fe, and the proportion varied with the soil physical and chemical properties, such as soil pH, soil water content, etc. For the impact of MOF on soil pH, previous studies showed that the MOF caused a significant pH decline [22]. This result was probably attributed to the difference in MOF structures. In our study, single crystal analysis result shows that the MOF contains only Fe^3+^, possible microbial degradation of MOF based on oxalate-carbonate pathway can increase soil pH. However, previous related research suggests that about a third of the Fe in the OPA-MOF is present as Fe^2+^. Hence, because of a higher redox potential of Fe^2+^ oxidation to Fe^3+^ in comparison to NH_4_^+^ nitrification, Fe^2+^ oxidation is a preferred reaction, resulting in release of two protons for each oxidized Fe-atom [39]. Thus, this chemical process probably leads to a decline in soil pH.

In summary, considering the feasibility of the synthetic procedure and the breakdown and degradation of the MOF material in soil, we gradually evaluated the degradation rate and nutrient release levels of the MOF compound. The results show that the MOF can provide nutrients continuously and efficiently throughout the growth period of wheat. Therefore, this material can potentially be applied in controlled-release fertilizers.

## 5. Conclusions

This study shows that MOF degraded behavior in the crop field. The ATR-FTIR and LIBS spectra of MOF underwent significant changes during the degradation process. In addition, the SEM images show that the MOF degraded and peeled off in layers. Additionally, Our MOF degraded by 50.9% throughout the wheat growth period. Soil temperatures were found to have a great impact on the degradation of the MOF, with the degradation rate increasing as the soil temperature increased. Due to the nutrient release levels and efficiencies of the MOF, it can be utilized in controlled-release fertilizers.

## Figures and Tables

**Table 1:** Experiment soil nutrients.

Parameters	pH	Organic Matter (g/kg)	Total-N (g/kg)	NH_4_^+^-N (mg/kg)	NO_3_^−^-N (mg/kg)	Available P (mg/kg)	Available K (mg/kg)	Available Fe (mg/kg)
Value	6.72	18.23	1.48	25.36	32.26	22.25	182.5	6.48
